# Programmable DNA hydrogel provides suitable microenvironment for enhancing autophagy-based therapies in intervertebral disc degeneration treatment

**DOI:** 10.1186/s12951-023-02109-5

**Published:** 2023-09-28

**Authors:** Song Qingxin, Jiang Kai, Zheng Dandan, Jin Linyu, Chen Xiuyuan, Feng Yubo, Wang Kun, Han Yingchao, Chen Hao, Song Jie, Chen Zhi, Shen Hongxing

**Affiliations:** 1 Department of Spine Surgery Renji Hospital, Shanghai JiaoTong University School of Medicine, 160 Pujian Road, Shanghai, 200127 P. R. China; 2 Department of Ophthalmology and Vision Science, Ear, Nose and Throat Hospital, Shanghai Eye, Fudan University, Shanghai, China; 3 Department of Orthopedics, Shanghai Key Laboratory for Prevention and Treatment of Bone and Joint Diseases, Shanghai Institute of Traumatology and Orthopedics, Ruijin Hospital, Shanghai Jiaotong University School of Medicine, Shanghai, P. R. China; 4 Department of Instrument Science and Engineering, School of Electronic Information and Electrical Engineering, Shanghai Jiao Tong University, Shanghai, 200240 China; 5 The Cancer Hospital of the University of Chinese Academy of Sciences, Hangzhou, 310022 Zhejiang China

**Keywords:** Intervertebral disc degeneration, Gene therapy, DNA hydrogel, Autophagy, Extracellular matrix

## Abstract

The pathogenesis of intervertebral disc degeneration (IVDD) is attributed to metabolic dysregulation within the extracellular matrix and heightened apoptosis of nucleus pulposus cells (NPC). Therefore, a potential therapeutic strategy for managing IVDD involves the reestablishment of metabolic equilibrium within the extracellular matrix and the suppression of excessive myeloid cell apoptosis. The microRNA, miR-5590, displays marked differential expression in degenerative nucleus pulposus (NP) tissues and exerts a direct influence on the regulation of DDX5 expression. This, in turn, modulates mammalian target of rapamycin (mTOR) phosphorylation, thereby impacting autophagy and apoptosis. However, ensuring the smooth delivery of miRNA to a specific injury site poses a significant challenge. To address this issue, a multifunctional DNA hydrogel was developed and subsequently loaded with miR-5590 via spherical nucleic acids (SNAs) for the treatment of IVDD. The hydrogel, which exhibits versatility, has the potential to be administered through injection at the site of injury, resulting in a consistent and prolonged release of miR-5590. This leads to the creation of a genetic microenvironment within the NP, which triggers the onset of autophagy in NPCs and subsequently suppresses apoptosis. As a result, this process regulates the metabolic equilibrium within the extracellular matrix, thereby impeding the in vitro and in vivo progression of IVDD. The amalgamation of miRNAs and biomaterials offers a promising therapeutic strategy for the management of IVDD in clinical settings.

**Supplementary Information:**

The online version contains supplementary material available at 10.1186/s12951-023-02109-5.

## Introduction

Intervertebral disc degeneration (IVDD) is a prevalent musculoskeletal degenerative condition that is characterized by lower-back pain, disability, and a substantial socioeconomic burden worldwide [1, 2]. The current clinical management of IVDD primarily focuses on pain management in the early stages and surgical intervention in advanced stages [3]. However, these approaches have limited efficacy in halting the pathological progression of the disease. The administration of these treatments is concomitant with diverse adverse effects, such as an augmented susceptibility to complications related to the gastrointestinal tract and antiplatelet activity, an elevated probability of necessitating revision surgeries, a reduction in mechanical characteristics, and degeneration of contiguous intervertebral discs (IVDs) [4]. Consequently, it is crucial to formulate therapeutic strategies that target the prevention or suppression of the pathological advancement of IVDD.

Despite continued research efforts, the exact pathogenesis of IVDD remains elusive. Nevertheless, it is widely accepted that the most plausible mechanism involves an imbalance between anabolic and catabolic functions within the extracellular matrix (ECM) [5]. Disc cells typically sustain biological activities by producing ECM, which consists of collagen fibrils and proteoglycan [6]. IVDD is typified by the dysfunction and hypocellularity of the gelatinous nucleus pulposus (NP) tissue, which plays a crucial role in maintaining the structural stability and biomechanical balance of the spine [7, 8]. The progression of IVDD is exacerbated by heightened inflammation and excessive production of degradative enzymes, which further degrade the extracellular matrix (ECM) and cause mechanical alterations, ultimately resulting in disc structural malfunction, including loss of disc height, annulus fibrosus (AF) fissures, and decreased NP substance. Furthermore, these factors contribute to the escalated apoptosis of cells within the intervertebral disc (such as nucleus pulposus cells, NPCs) [9].

The emergence of molecular biology and genomics has led to the discovery of microRNAs (miRNAs) as crucial regulators of gene expression networks in vivo. These naturally occurring endogenous regulatory molecules are small double-stranded RNAs, typically measuring 19–22 bp in length [10]. Through strict or loose matching mechanisms, miRNAs can modulate the translation and degradation of target gene mRNAs, thereby inducing a systematic alteration in the expression of a series of genes and ultimately influencing cell phenotype [11]. In addition, miRNAs can be regulated by various transcription factors and signal transduction pathways *in vivo.* Recently, the miRNA mir-21 was found to be significantly upregulated in IVDD and promoted aberrant differentiation of the NP by inhibiting PTEN expression post-transcriptionally [12]. In another study, miR-155 expression was found to be significantly downregulated in IVDD. miR-155 specifically targets the 3’ untranslated region (UTR) of genes encoding apoptosis-related molecules FADD and caspase-3, and the overexpression of mir-155 leads to downregulation of these genes, thereby inhibiting the apoptosis of NPCs [13]. Thus, microRNAs may be important for the development of IVDD, and altering the expression of some miRNAs may effectively slow down or inhibit IVDD pathogenesis. In a prior investigation, Wu et al. discovered that miR-5590-3p has the capacity to directly target the 3′ UTR of DEAD (Asp-Glu-Ala-Asp) box helicase 5 (DDX5) [14]. DDX5 is recognized as a nuclear protein with multiple biological functionalities, and its expression and modulation are pertinent to the pathogenesis of various ailments and are subject to the influence of diverse signaling cascades [15]. DDX5 can regulate autophagy by modulating the phosphorylation of mammalian target of rapamycin (mTOR) [16]. Manipulating the expression of specific miRNAs, such as miR-5590, may represent a viable approach to impede or decelerate the progression of the disease.

However, the precise delivery of miRNAs to the injury site and their subsequent response to the injury pose a significant challenge. The cellular uptake of exogenous miRNAs results in their degradation within the lysosome, thereby reducing the effective concentration of therapeutic miRNAs [17]. To circumvent lysosomal degradation, plasmids or viral vectors are frequently utilized to overexpress miRNAs in cancer cells [18]. Nonetheless, the practical effectiveness of this approach in clinical settings is restricted due to the significant collateral damage incurred by non-cancerous cells and the intricate multistage process involved [18, 19]. In this regard, spherical nucleic acids (SNAs), nanostructures consisting of densely arranged nucleic acids on the surface of microspheres, have emerged as a promising delivery vehicle. The negative charge on the surface of SNAs enables the surface nucleic acid to persist for an extended period without exhibiting any discernible gene expression or toxicity. Gold nanoparticles (AuNPs) are frequently utilized as the core for SNAs due to their densely loaded oligonucleotide surfaces, which facilitate the effective delivery of miRNAs for gene therapy. Nevertheless, the efficacy of targeted therapy may be compromised in certain deep tissues, such as the IVD, due to the potential for leakage and spread. Therefore, achieving sustained delivery of miRNAs by SNAs requires the use of an injectable biomaterial that exhibits sufficient mechanical strength and uniform nanoparticle loading capacity. The unique properties of DNA hydrogels, including biocompatibility, porosity, sequence programmability, and tunable multifunctionality, have garnered significant attention in the fields of bioanalysis and biomedicine [20]. Moreover, the incorporation of 3D scaffolds in DNA hydrogels has resulted in improved mechanical stability and increased attachment points, making them an effective immobilization medium for nanoparticles and molecular constituents. As a result, DNA hydrogels have emerged as a promising candidate for injectable therapies in the treatment of IVDD.

**Fig. 1:**
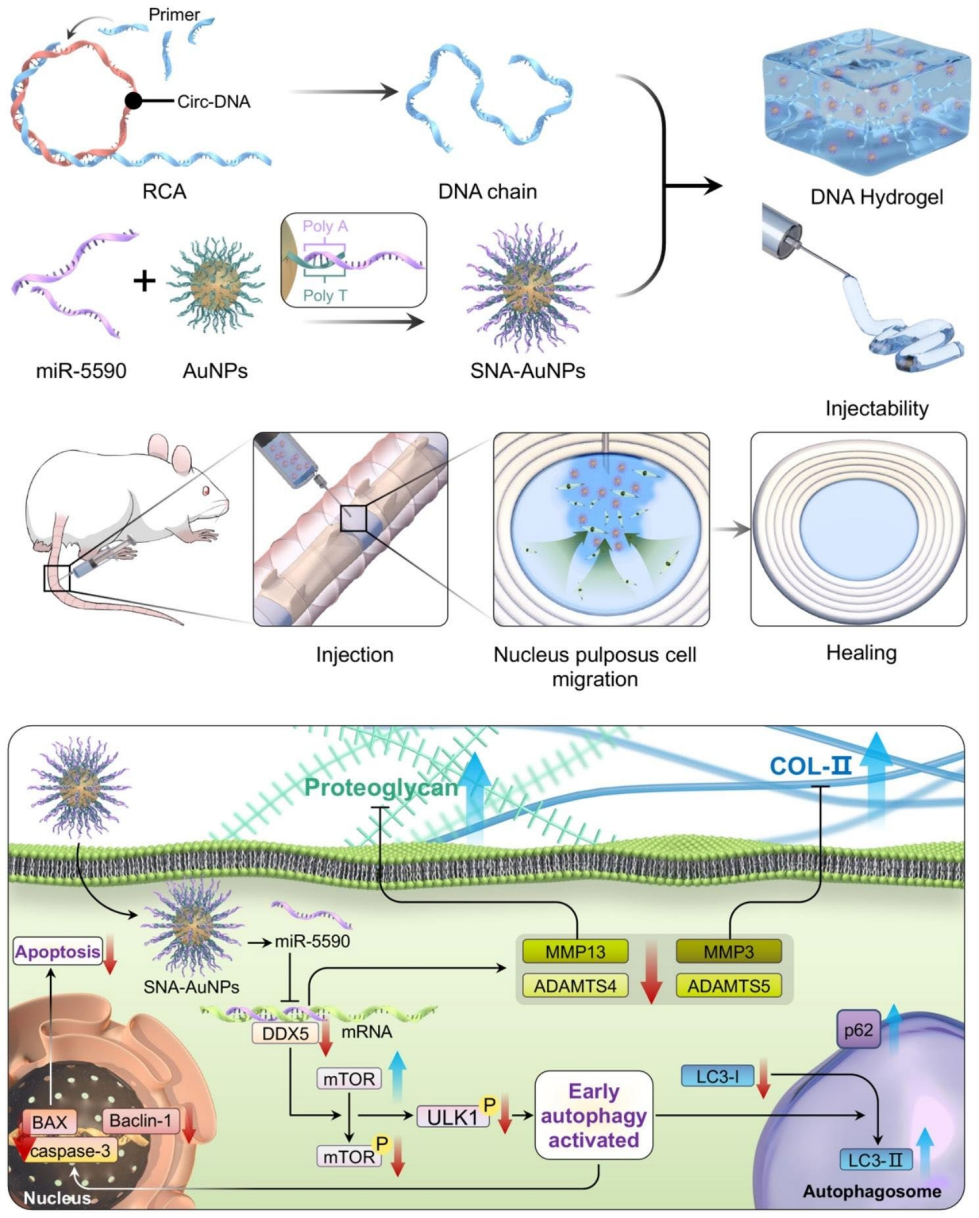
Schematic representation of the fabrication process used to prepare the miR-5590-SNA@DNAgel.

In this study, a multifunctional, injectable, self-healing DNA hydrogel was synthesized as a carrier for SNAs coated with miR-5590 (miR5590-SNA@DNAgel) (Fig. 1). The composite hydrogel demonstrated excellent nanoparticle loading uniformity and targeted implantation ability within the microenvironment of IVDs when administered via a micro syringe. The hydrogel enables the regulated and extended discharge of potent therapeutic miRNAs, thereby mitigating inflammation-induced harm and enhancing inflammatory microenvironments within intervertebral discs (IVDs) for improved therapeutic results. The miR5590-SNA@DNAgel effectively suppressed the inflammatory cascade, which had been initiated by the activation of inflammatory pathways. This, in turn, led to the regulation of both apoptosis and autophagy, ultimately resulting in the reversal of metabolic disorders of the extracellular matrix. The implementation of miR5590-SNA@DNAgel in the rat IVDD model enabled the imposition of mechanical stress, resulting in reduced MMP expression, expedited NP regeneration, and the reorganization and fortification of the ECMs in the deteriorated IVDs.

## Results and discussion

### Fabrication and characterization of miR5590-SNA@DNAgel

**Fig. 2:**
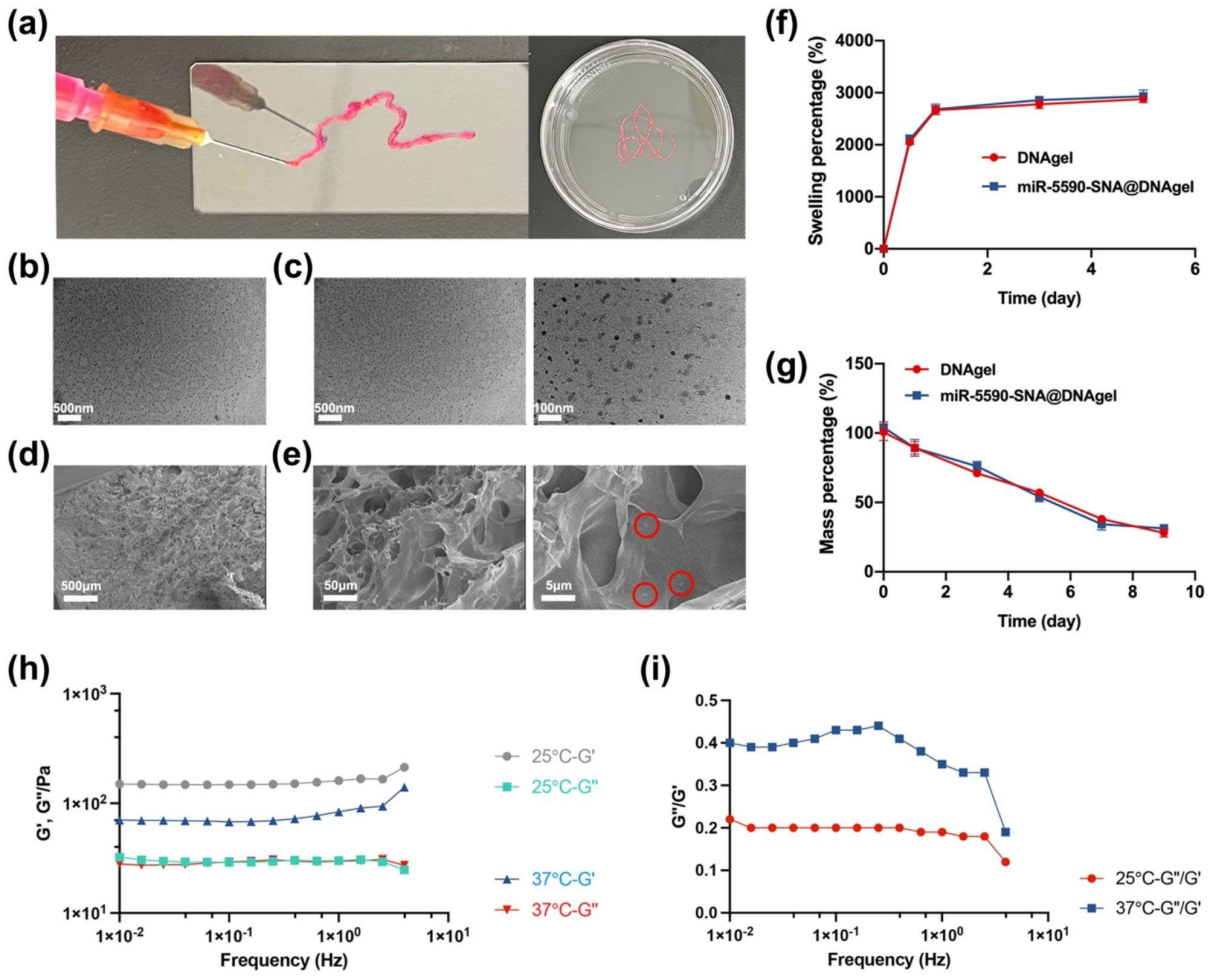
Properties of the miR-5590-SNA@DNAgel. (**a**) Photograph of the injectable hydrogel. (**b**,**c**) SEM images of (b) SNA nanoparticles and (c) miR-5590 loaded SNA nanoparticles. (**d**,**e**) SEM images of (d) DNAgel and e) miR-5590-SNA@DNAgel (miR-5590-SNA was marked by red circle). (**f**) Swelling percentage. (**g**) Degradation percentage. (**h**) Storage modulus (G′) and loss modulus (G′′) of miR-5590-SNA@DNAgel. (**i**) Loss tangent of miR-5590-SNA@DNAgel.

To treat IVDD, a DNA hydrogel can be used as a carrier to load drugs for intervertebral space injection, which can serve as a minimally invasive therapy to significantly reduce the trauma caused by treatment and reduce leakage at the injection site. In the present study, DNA hydrogel was successfully constructed that can smoothly pass through a 1-mL syringe and maintain a colloidal shape after a minimally invasive injection into deep tissue (Fig. 2a). The results of scanning electron microscope (SEM) showed that the SNA particles exhibited a uniform, spherical, hollow structure (Fig. 2b), and the microstructure of the SNA particles did not change significantly after loading with miR-5590 (Fig. 2c). The SEM images of the DNA hydrogel revealed a loose, porous-like structure after lyophilization (Fig. 2d), and each pore was interconnected; the microstructure was unchanged by the addition of miR5590-SNA, which aggregated into microparticles when adsorbed to the hydrogel (Fig. 2e). The porous cavities of the DNA hydrogel have the potential to increase drug loading capacity and provide a suitable microenvironment for the function of miR-5590-SNA in situ, effectively creating a gene-hydrogel microenvironment.

Next, we assessed the swelling capacity of the DNA hydrogel and found that contact with phosphate-buffered saline (PBS) resulted in a rapid increase in mass to more than 2500% of the original mass (Fig. 2f), which was maintained for a week. Therefore, the DNA hydrogel exhibited an excellent water absorption capacity with good water maintenance, showing promise for absorbing water-soluble drugs and maintaining moisture inside the IVD. In addition, the swelling curve of the miR-5590-SNA@DNAgel indicated good water absorption and maintenance properties.

The degradability of biomaterials is an important property because such materials need gradually degrade for supporting tissue repair and regeneration [21]. To simulate the renewal of body fluids, in vitro degradation experiments using PBS were performed to simulate the pH values of body fluids and changing the PBS solution every two days. The DNA hydrogel was maintained for the first three days, with degradation accelerating after five days and only 20% of the original mass remaining after nine days (Fig. 2g). miR-5590-SNA can thrive in the microenvironment provided by the early DNA hydrogel owing to its long half-life and slow degradation rate. Under the regulation by miR-5590-SNA, the synthesis of ECM by NPCs increases, and the DNA hydrogel is degraded; this reduces the impact on the synthesis of ECM and promotes the regeneration of NP tissue. In addition, the hydrogel exhibits a fluid-like behavior at 37 °C, enabling its injectability. Conversely, within the disc, it assumes a solid-like colloidal state, facilitating efficient retention of miR-5590-SNA (Fig. 2h, i).

In conclusion, we have fabricated an injectable DNA hydrogel microenvironment with high porosity, biodegradability, and strong water absorption properties. A gene-hydrogel microenvironment was formed in situ by loading the hydrogel with miR-5590-SNA, and the microstructure of this microenvironment was key to preventing leakage of miR-5590-SNA while also providing a suitable environment for it to function.

### Cytocompatibility assessment and cellular uptake of mir-5590-SNA in vitro

**Fig. 3:**
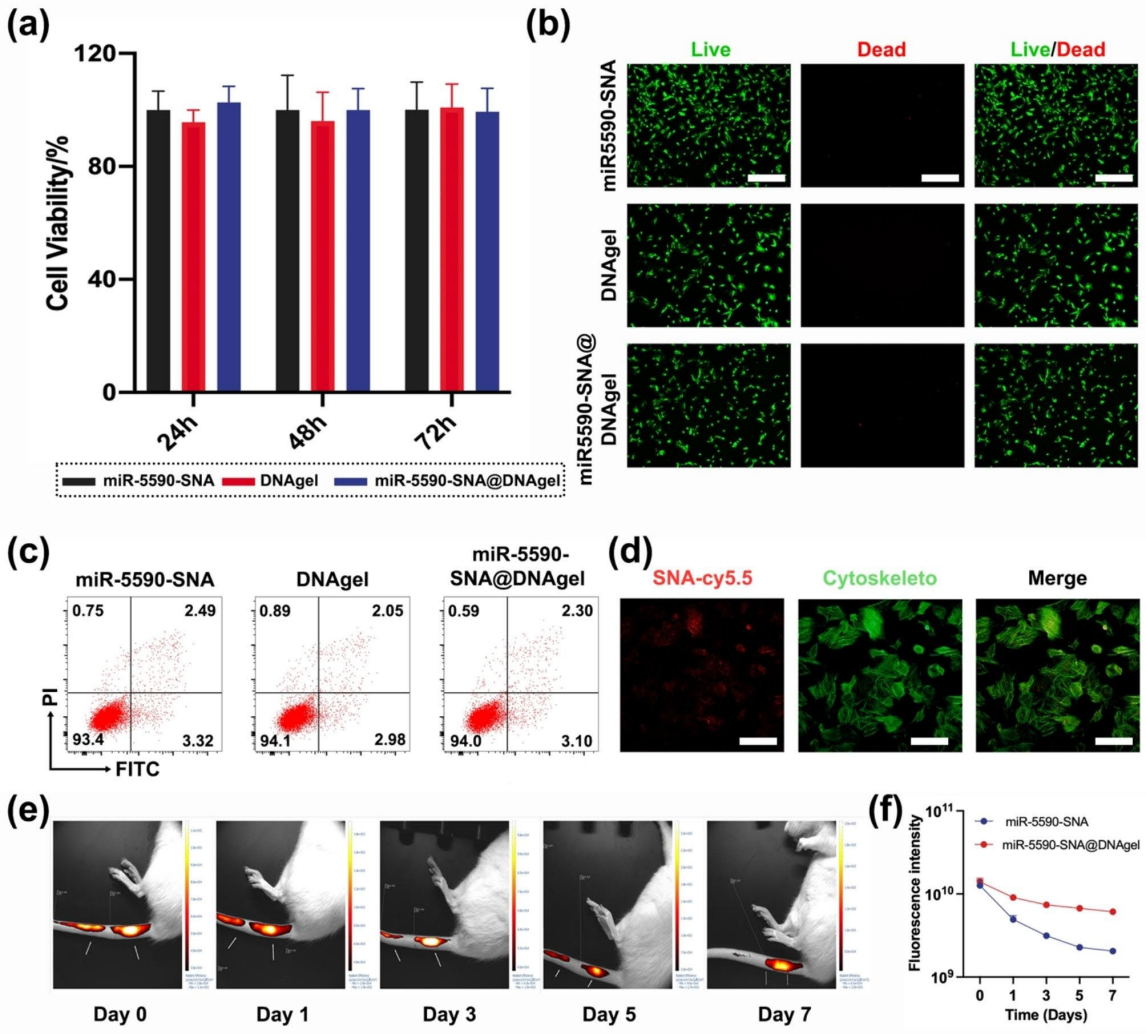
Biological study of miR-5590-SNA@DNAgel. (**a**) Cytotoxicity of miR-5590-SNA@DNAgel. (**b**) Live/dead staining of NPCs co-cultured with miR-5590-SNA@DNAgel. Scale bar: 200 μm. (**c**) Cell apoptosis were carried out after incubation with miR-5590-SNA@DNAgel. (**d**) Cy5.5-labeled SNA cellular uptake assay. Scale bar: 50 μm. (**e**) The images of in vivo retention test. (**f**) The fluorescence intensity changes with time of miR-5590-SNA and miR-5590-SNA@DNAgel. Data are expressed as average ± SD (n = 3)

Cell biocompatibility is an essential characteristic of biomaterials [22]. In the present study, cell biocompatibility responses to miR-5590-SNA@DNAgel were detected by the CCK-8 assay. After 24, 48, and 72 h of treatment, miR-5590-SNA@DNAgel did not display any cytotoxicity (Fig. 3a). In addition, the images of live/dead staining assay showed > 95% cell viability, suggesting the excellent biocompatibility of the miR-5590-SNA@DNAgel (Fig. 3b). Furthermore, NPCs treated with miR-5590-SNA@DNAgel did not undergo early apoptosis when compared to other groups (Fig. 3c). Based on the above results, the novel gene-hydrogel system demonstrated substantial biocompatibility with low cytotoxicity and may be widely applied in tissue regeneration. Delivery of miRNAs is essential for successful treatment [23]. Thus, cellular uptake assays results showed that SNA efficiently delivered miRNAs into the cytoplasm of NPC cells (Fig. 3d), laying the foundation for the post-transcriptional regulation of miRNAs.

### miR-5590-SNA@DNAgel effectively prevented leakage

The IVD is an important load-bearing structure in the vertebrate spine that connects two adjacent vertebral bodies; hence, the IVD is continuously active in different directions [24]. The use of simple liquid drugs for intervertebral space injection in the treatment of IVDD is prone to reverse leakage along the needle track and subsequent spread to adjacent tissues owing to continuous stress and extrusion of the injected fluid, thereby reducing the efficacy of the drug and causing serious age-related complications [25]. With the development of biological tissue engineering, drugs loaded into biomaterials can effectively prevent the leakage of drugs following injection [26]. We compared leakage of miR-5590-SNA following injection with or without the DNA hydrogel. By injecting near-infrared (NIR) dye-labeled miR-5590-SNA and NIR dye-labeled miR-5590-SNA@DNAgel into the intervertebral space of rats, we found that the use of the DNA hydrogel effectively prevented leakage, which was limited to around the intervertebral space; conversely, cord-like fluorescence areas were observed in the group treated without DNA hydrogel, suggesting drug leakage. In addition, the fluorescence intensity observed in the DNA hydrogel group remained at 50% at 1-week post-drug injection (Fig. 3e). The time-fluorescence intensity curve showed that the curve of miR-5590-SNA@DNAgel group was higher than that of miR-5590-SNA group (Fig. 3f). The aforementioned findings demonstrate that the utilization of miR-5590-SNA@DNAgel exhibited a commendable anti-leakage efficacy throughout the injection procedure, thereby significantly mitigating potential adverse reactions of the medication and augmenting its retention rate within the organism. Consequently, this intervention effectively enhances the longevity of the drug’s therapeutic effect.

### miR-5590-SNA@DNAgel regulated the autophagic and apoptotic of NPCs

The core pathological mechanism underlying many diseases in the human body is the breakdown of the anabolic balance of the ECM in tissues [27]. The early stage of NP degeneration is characterized by the occurrence of dysfunctional NPCs and commonly involves NPC death [28]. Many studies have demonstrated that the most common types of NPC death include autophagy and apoptosis [29]. Autophagy is a unique life phenomenon widely present in eukaryotic cells and is closely related to cell growth, proliferation, and apoptosis [30]. Autophagy has long been considered a self-protective response of cells in the face of harmful stimuli such as nutrient deprivation, hypoxia, and oxidative stress. Autophagy can selectively scavenge damaged or excess oxygen free radicals, peroxisomes, mitochondria, and other substances in cells and reduce the accumulation of abnormal proteins and organelles to maintain the self-homeostasis of cells [31]. A variety of autophagy-related genes (Atg) are involved in the formation of autophagy, such as p62, LC3, Beclin-1, and ULK1 [32]. p62, also known as SQSTM1, is involved in the regulation of autophagosomes, is degraded during the middle and late stages of autophagy, and its expression negatively correlates with autophagic activity [31]. LC3 (also name Atg8), has two forms, LC3-I and LC3-II, respectively, and is a necessary substance for the formation of the autophagosome bilayer membrane structure [33]. LC3-I was primarily located in the cytoplasm and localized to autophagosomes after being transported and modified into LC3-II following the activation of autophagy; thus, LC3-II/LC3-I serves as an autophagy marker [34]. The autophagy-related protein, Beclin-1, also known as Atg6, is important in autophagosome formation, maturation, and endocytosis, which mediates changes in the bilayer membrane structure after forming complexes with a variety of substances [35]. Studies have shown that early autophagy can inhibit cell apoptosis and thus reduce cell death [36].

In recent years, with the deepening of autophagy research, researchers have gradually revealed some signaling pathways associated with autophagy. For example, signaling pathways such as phosphatidylinositol 3-kinase (PI3K)/protein kinase B (PKB/Akt)/mammalian target of rapamycin (mTOR), mitogen-activated protein kinase (MAPK), and adenylate activated protein kinase (AMPK) or the relationship between related kinases and autophagy have been demonstrated [37]. Among them, the PI3K/Akt/mTOR signaling pathway is a relatively well-studied one, which plays a crucial role in the molecular mechanism of autophagy, signal regulation, and cell growth, proliferation, differentiation, and apoptosis. mTOR is currently recognized as a “monitor” and “gatekeeper” in the process of autophagy, and mTOR is an autophagy negatively regulated kinase, that is, activation of mTOR inhibits autophagy, and conversely, inhibition of mTOR induces autophagy [38].

**Fig. 4:**
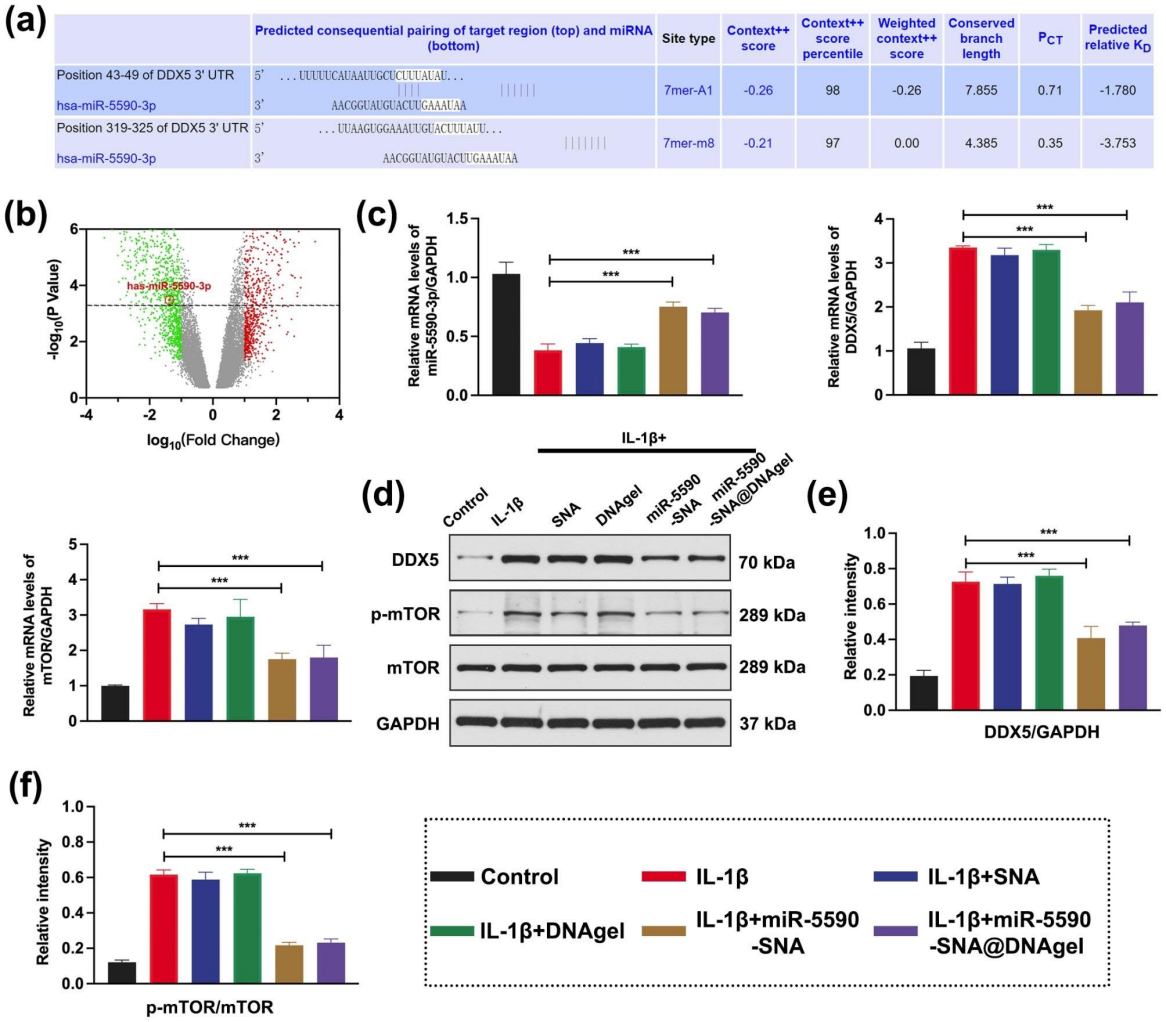
miR-5590 regulates DDX5 and mTOR expression. (**a**) Analysis of DDX5 and miR5590 binding sites. (**b**) The volcano plot of differential expression of miRNAs in IVDD. Red circles denote miR-5590 under-expressed in degenerative intervertebral disc. (**c**) Real-time PCR detection of gene expression in NPCs. (**d**) Western blotting data showing the levels of the expression of DDX5, p-mTOR, and mTOR. (**e, f**) Western blotting data of the levels of the protein in (**d**). Data are expressed as average ± SD (n = 3). **P < 0.01; ***P < 0.001

In the Gene Expression Omnibus database (GSE116726), miRNA expression differences in degenerative NP tissues were investigated [39]. The online tool TargetScan (targetscan.org/vert_72/) was utilized to identify a potential binding site of miR5590, consisting of 7 bases, on the 3’ UTR of DDX5 (Fig. 4a). In comparison to the controls, miR-5590 was under-expressed in degenerative NP tissues (Fig. 4b). Downregulation of miR-5590 expression in NPCs following IL-1β treatment, while the expression of miR-5590 recovered after combinatorial IL-1β + miR-5590-SNA or IL-1β + miR-5590-SNA@DNAgel treatment, indicating that miR-5590 may be associated with downregulated expression of DDX5 (Fig. 4c-f). This result suggests that DDX5 may be a target gene of miR-5590 in NPCs.

**Fig. 5:**
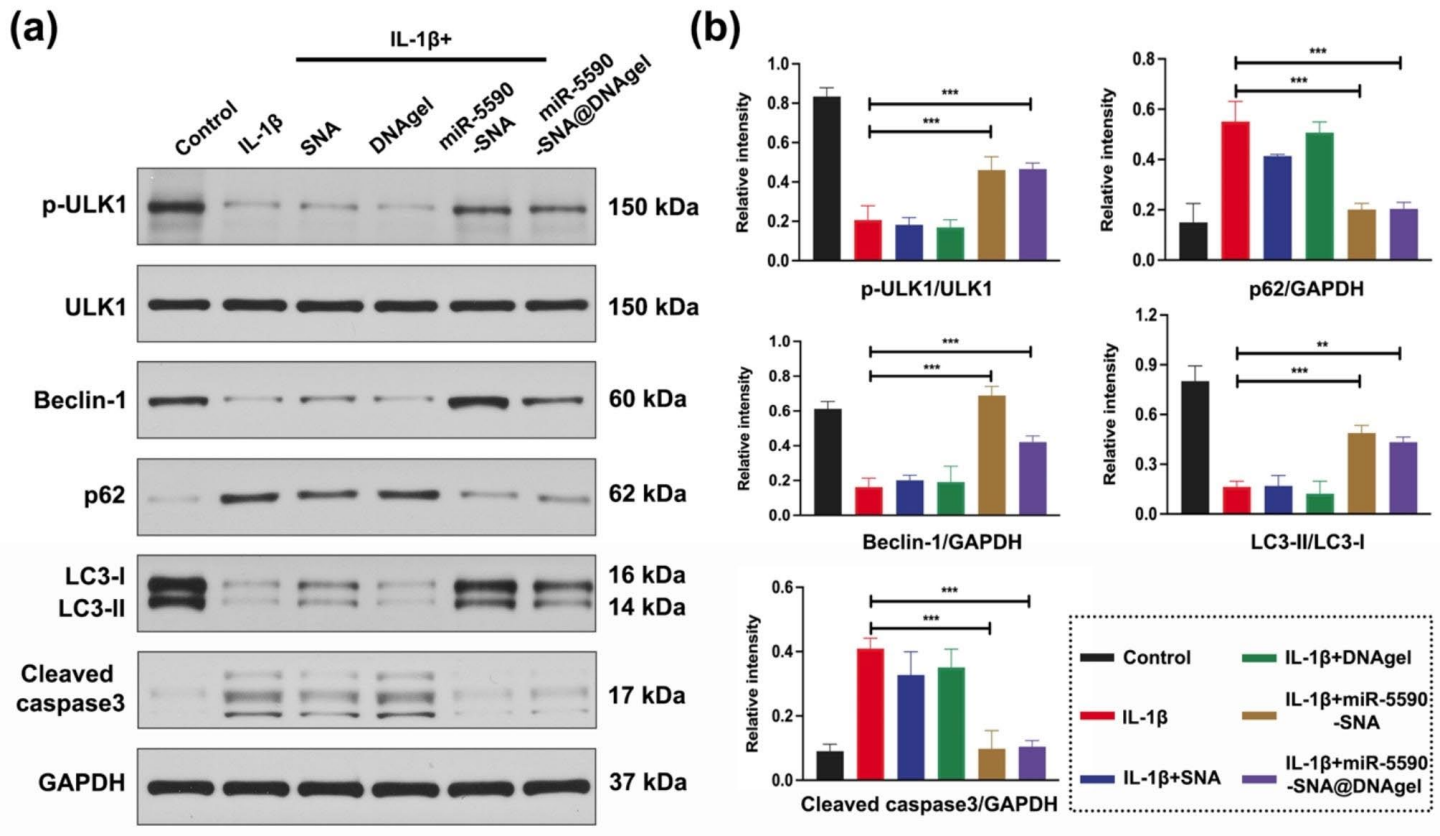
Western blotting detection of miR5590-SNA@DNAgel regulates the protein expression related to autophagy and apoptosis in NPCs. (**a**) Western blotting data showing the levels of the expression of p-ULK1, ULK1, Beclin-1, p62, LC3-I, LC3-II, and anspase3. (**b**) Western blotting data of the levels of the protein in (**a**). Data are expressed as average ± SD (n = 3). *P < 0.05; **P < 0.01; ***P < 0.001

Further studies suggested that the phosphorylation of mTOR was inhibited by the downregulation of DDX5, which increased the expression of pULK1 (Fig. 5a-b), thereby initiating autophagy. Moreover, it was observed that the treatment of miR-5590-SNA induced upregulation of Beclin-1, downregulation of p62, and indicative upregulation of LC3-II/LC3-I in NPCs (Fig. 5a-b). In this study, we found that DDX5 expression was downregulated after miR-5590-SNA@DNAgel treatment, promoting the occurrence and development of early autophagy in NPCs. At the same time, we found that the apoptosis-related protein for caspase-3 were downregulated (Fig. 5a-b), indicating that early autophagy in NPCs inhibited apoptosis and reduced cell death, thus playing a protective role in NPCs during degeneration.

### miR-5590-SNA@DNAgel regulated the anabolic balance of the ECM in NPCs

**Fig. 6:**
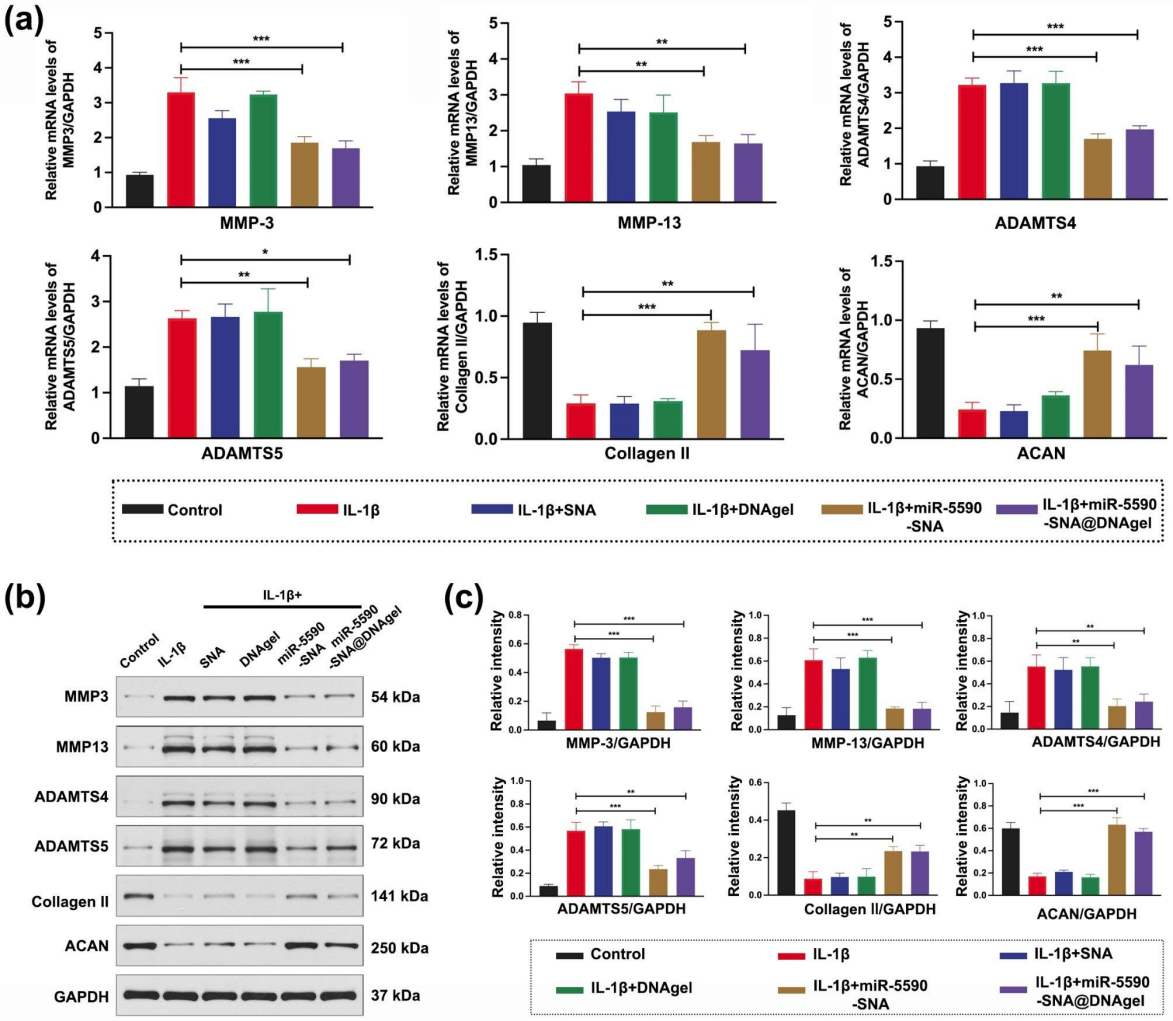
NPCs synthesis/catabolism balance induced by miR-5590-SNA@DNAgel. (**a**) Real-time PCR detection of gene expression in NPCs. (**b**) Western blotting data showing the levels of the expression of MMP3, MMp13, ADAMTS4, ADAMTS4, Collagen II, and ACAN. (**c**) Western blotting data of the levels of the protein in (**b**). Data are expressed as average ± SD (n = 3). *P < 0.05; **P < 0.01; ***P < 0.001

During the process of degeneration, excessive decomposition and destruction of the ECM in the microenvironment of the NP tissue disrupt the anabolic balance of the ECM, resulting in decrease in the water retention capacity of NP tissue, causing further shrinkage of NP tissue and acceleration of IVDD pathogenesis [40]. ECM catabolism in the NP primarily involves the ADAMTS and matrix metalloproteinases (MMP) families, while the ECM is mainly composed of type II collagen and proteoglycans [41, 42]. MMP-3 is a gelatinase that degrades denatured collagen and gelatin. MMP-13 is an interstitial collagenase that degrades many collagens and most polysaccharide molecules. We found that the expression of ADAMTS4, ADAMTS5, MMP3, and MMP13 was downregulated, while the expression of ECM components, especially type II collagen and Aggrecan (ACAN), was upregulated after miR-5590-SNA treatment (Fig. 6a). miR-5590-SNA@DNAgel negatively regulated the expression of ADAMTS and MMPs, thereby reducing ECM degradation and increasing the ECM content surrounding NPCs (Fig. 6b-c). Thus, by regulating the anabolic balance of the ECM in the NP, miR-5590-SNA@DNAgel reversed the anabolic imbalance and inhibited IVDD pathogenesis. These results demonstrate that injection of a DNA hydrogel loaded with miR-5590-SNA into the intervertebral space during the early stage of IVDD pathogenesis regulates NPC autophagy, apoptosis, and ECM synthesis/decomposition. Balanced catabolism conferred by the in-situ construction of a gene-hydrogel microenvironment in the IVD improves the microenvironment of NP tissue and promotes NP regeneration during IVDD treatment.

**Fig. 7:**
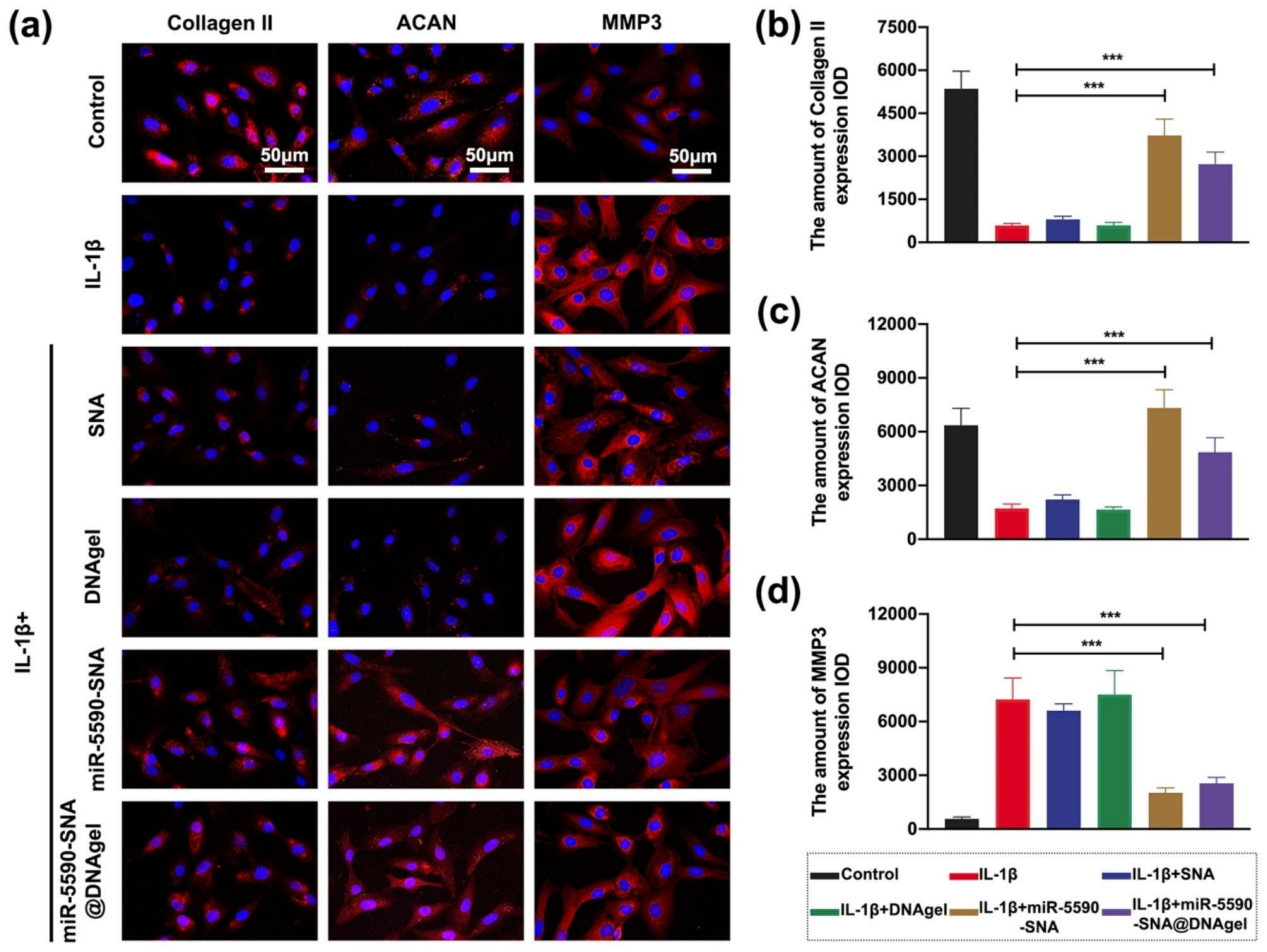
Immunofluorescent staining of NPCs on miR5590-SNA@DNAgel. (**a**) Collagen II, ACAN, and MMP3 immunofluorescent staining of NPCs: from left to right panel: Collagen II (red), ACAN (red), and MMP3 (red) (OCN); DAPI (blue). Scale bar: 50 μm. (**b-d**) The amount of (**b**) Collagen II, (**c**) ACAN, and (**d**) MMP3 expression IOD. Data are expressed as average ± SD (n = 3). *P < 0.05; **P < 0.01; ***P < 0.001

Additionally, Collagen II, ACAN and MMP3 by immunofluorescence staining were determined, and the results showed that NPCs in the miR-5590-SNA and miR-5590-SNA@DNAgel groups expressed more Collagen II (red) and ACAN (red) than those in the IL-1β group (Fig. 7a-c). In contrast, less expression of MMP3 was observed in miR-5590-SNA and miR-5590-SNA@DNAgel groups than in the IL-1β group (Fig. 7a and d). These results were concordant with the WB and qRT-PCR results.

### miR-5590-SNA@DNAgel promoted intervertebral disc regeneration in vivo

**Fig. 8:**
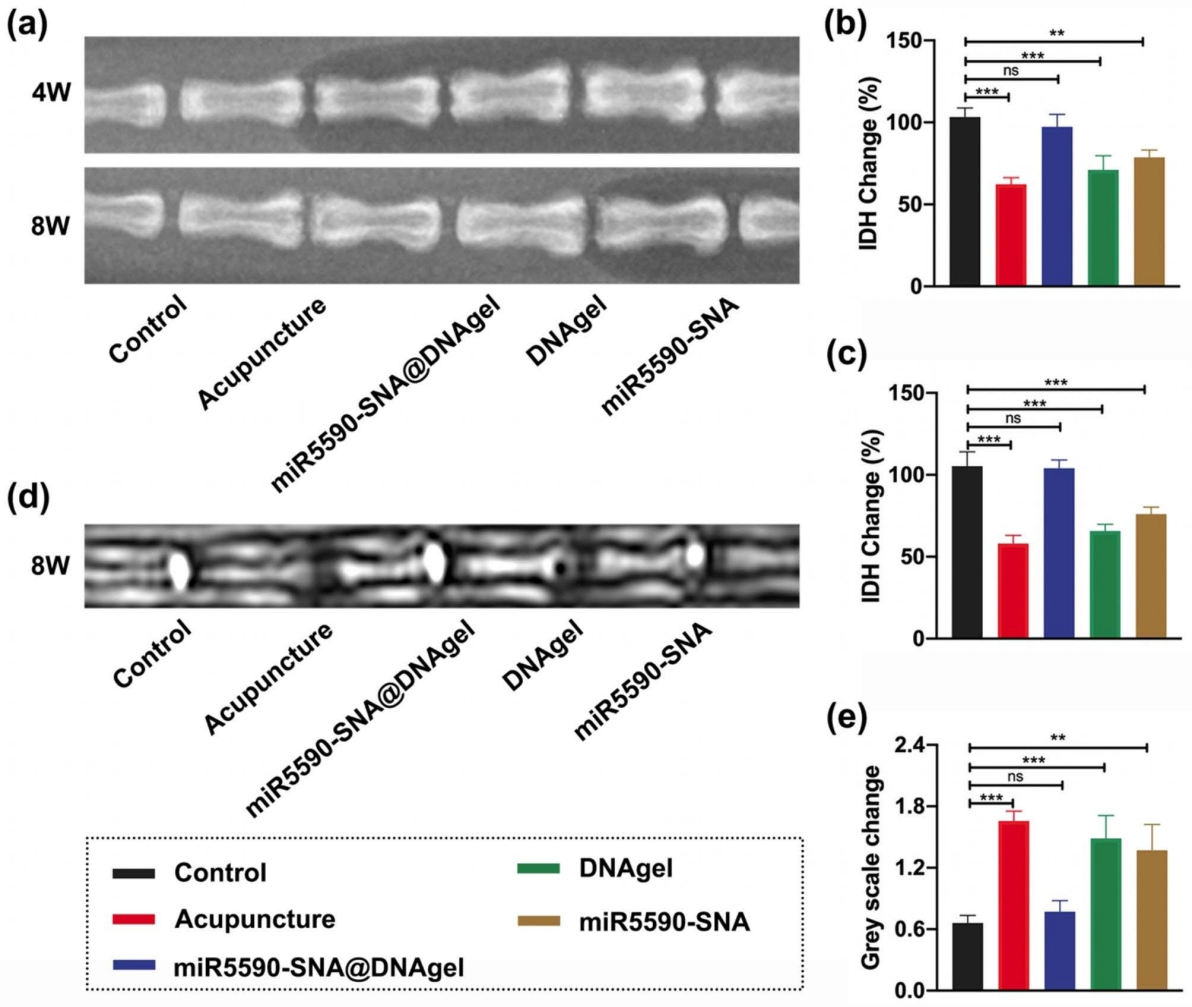
Imaging data of in vivo IVDD model. (**a**) The rat coccygeal vertebrae of different treatment group at 4- and 8-week after surgery was assessed by X-ray. (**b**, **c**) Intervertebral disc heights (IDH) of different groups at (**b**) 4-week and (**c**) 8-week. d) The rat coccygeal vertebrae of different treatment group at 8-week after surgery was assessed by MRI. (**e**) The gray scale changes of different treatment group at 8-week after surgery. Data are expressed as average ± SD (n = 5). **P < 0.01; ***P < 0.001

To further investigate the effect of miR-5590-SNA@DNAgel treatment in vivo, a rat disc degeneration acupuncture model was used, as previously reported [43]. Radiographic and histological examinations of the IVD were performed at different time points to observe the internal condition of the IVD. Changes in ECM content in the IVD were estimated by measuring changes in the intervertebral space height between each vertebral body using radiography (Fig. 8a). Over time, the height of the intervertebral space of the degenerated discs in rats injected with miR-5590-SNA@DNAgel gradually returned to normal, similar to the trend observed in the control group. While the height of the intervertebral space of the segments receiving acupuncture became gradually smaller over time, the IVDs developed IVDD after a period. Likewise, changes in the intervertebral space of segments treated with the DNA hydrogel alone were similar to those in the acupuncture model, indicating that DNA hydrogel treatment alone did not have a significant effect on IVDD pathogenesis (Fig. 8a-c). Conversely, in the miR-5590-SNA treatment group, the height of the intervertebral space recovered over time but still exhibited a gap compared to the normal control group (Fig. 8a-c); this may have been associated with leakage and limited duration of action after miR-5590-SNA injection. Notably, there was no significant difference between the discs treated with miR5-590-SNA@DNAgel and the control group at any time point; therefore, the in-situ gene-hydrogel microenvironment formed by miR-5590-SNA@DNAgel injection into the intervertebral space regulated NPC death and ECM metabolism, thereby increasing the regenerative capacity of the degenerated IVD and providing unique advantages in restoring the height of the intervertebral space of degenerated IVDs.

We performed magnetic resonance imaging (MRI) of the experimental rats at 8 weeks. In the MRI images, the gray-level inside the disc reflects the water content, with a higher gray-level corresponding to a lower water content (Fig. 8d). Compared with preoperative animals, the water content of the caudal IVD after acupuncture surgery was significantly lower, while the water content of the IVD treated with miR-5590-SNA@DNAgel was slightly, but not significantly, reduced. The segmental IVDs treated with DNA hydrogel alone suffered a severe water loss comparable to that of the rat acupuncture model of IVDD (Fig. 8d-e). Partial loss of water inside the IVDs treated with miR-5590-SNA alone resulted in limited repair. These results indicate that miR-5590-SNA@DNAgel injection into the intervertebral space can effectively retain the water content of the degenerated IVD, helping to inhibit the pathogenesis of IVDD.

**Fig. 9:**
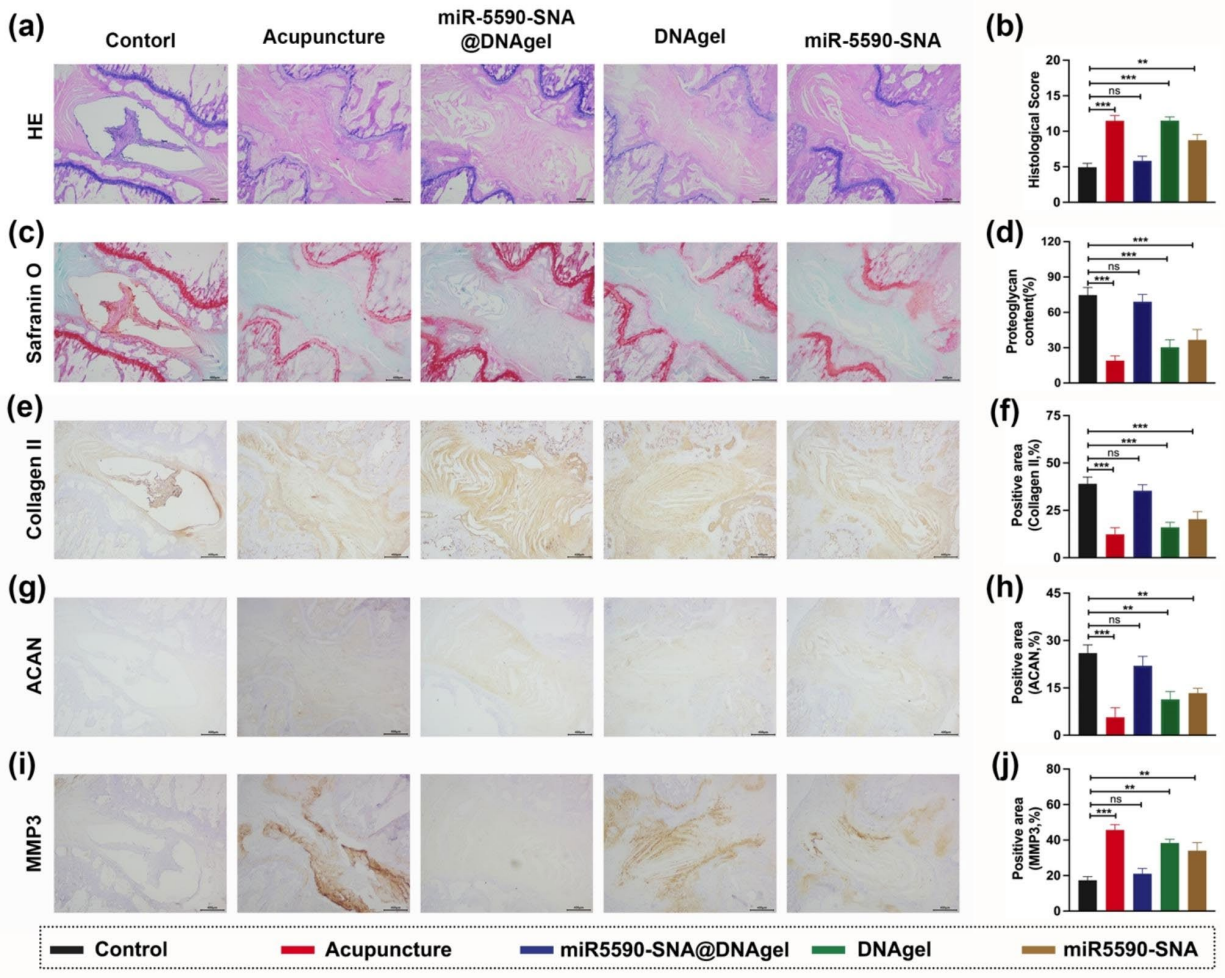
Histological and immunohistochemistry images of intervertebral disc in different groups. (**a**) The HE staining of intervertebral disc in different groups. Scale bar, 400 μm. (**b**) Histological scores of different groups. (**c**) The Safranin O staining of intervertebral disc in different groups. Scale bar, 400 μm. (**d**) The proteoglycan content of nucleus pulposus. (**e, f**) The immunohistochemistry staining (**e**) and quantification of positive area (**f**) of Collagen II. (**g, h**) The immunohistochemistry staining (**g**) and quantification of positive area (**h**) of Collagen ACAN. (**i, j**) The immunohistochemistry staining (**i**) and quantification of positive area (**j**) of Collagen MMP3. Scale bar, 400 μm. Data are expressed as average ± SD (n = 5). **P < 0.01; ***P < 0.001

To characterize the gene-hydrogel microenvironment constructed locally in situ after miR-5590-SNA@DNAgel injection into the intervertebral space and to explore the mechanisms maintaining the height of the intervertebral space and preserving the water content of the IVD, histological sections were performed on the IVDs of rats at 8 weeks post-surgery. The morphology of the NP and its demarcation from the AF were observed using hematoxylin and eosin (H&E) staining. H&E staining revealed that the morphology of the NP in the miR-5590-SNA@DNAgel group was significantly improved, and the boundary between the NP and AF tissues was clear and regular. However, the morphology of the NP in rats treated with the DNA hydrogel alone deteriorated, and the demarcation between the NP and AF tissues was blurred; a similar phenotype was observed in rats receiving acupuncture only (Fig. 9a). Although the demarcation between the NP and AF tissues was more regular in the miR-5590-SNA treatment group than in the acupuncture group, the degeneration rating remained relatively low. As previously described, the histological score was evaluated [44]. At 8 weeks after operation, the miR-5590-SNA@DNAgel group showed significantly lower histological scores than other groups, indicating the recovery of the AF and NP (Fig. 9b). Based on the Safranin O staining, the proteoglycan levels in the Acupuncture, DNAgel, and miR5590-SNA groups were decreased compared to those in the miR-5590-SNA@DNAgel group (Fig. 9c-d). Collagen II and ACAN should be present in sufficient quantity in a healthy nucleus pulposus in order to maintain its functional properties. As shown by immunohistochemistry staining, both miR-5590-SNA@DNAgel and control groups had normal levels of collagen II and ACAN. Nucleus pulposus collagen II was decreased and no ACAN expression was evident in the acupuncture, DNAgel, and miR-5590-SNA groups (Fig. 9e-h). In contrast, less expression of MMP3 was observed in miR-5590-SNA and miR-5590-SNA@DNAgel groups than in the IL-1β group. Comparatively, the control and miR-5590-SNA@DNAgel groups had lower MMP3 levels than that in the acupuncture, DNAgel, and miR-5590-SNA groups (Fig. 9i-j).

While the DNA hydrogel provides some support for the intervertebral space soon after treatment, it does not confer a sufficient long-term advantage in the treatment of IVDD because it degrades within one week of injection. In addition, although miR-5590-SNA can inhibit the apoptosis of NPCs and regulate the anabolic balance of the ECM in vitro, it is difficult to fully elucidate its function in vivo because it cannot be retained for an extended period. Under the action of the internal pressure of the IVD, miR-5590-SNA in solution is prone to leakage against the needle track after injection, resulting in a decrease in the amount of miR-5590-SNA that actually enters the IVD and reducing the efficacy of treatment. Fortunately, the miR-5590-SNA@DNAgel was able to form a gene-hydrogel microenvironment in the NP tissue inside the IVD, which can target and regulate ECM anabolic balance during IVDD pathogenesis, thus playing a key role in inhibiting IVDD.

## Conclusion

The present study involved the design and synthesis of a multifunctional DNA hydrogel possessing injectable, self-healing, biodegradable, and superabsorbent properties. This hydrogel was loaded with the gene-drug miR-5590-SNA for the treatment of IVDD. The injection of miR-5590-SNA@DNAgel resulted in the formation of a gene-hydrogel microenvironment in situ, which prevented the leakage of miR-5590-SNA from the target tissue. Furthermore, miR-5590-SNA exhibited significant efficacy in slowing down the pathogenesis of IVDD by directly regulating the autophagic and apoptotic processes of NPCs, thereby regulating the anabolic balance. Additionally, it has been observed that DNA hydrogels possess the ability to uphold the initial mechanical potency of the IVD and augment water retention, thereby furnishing support for the intervertebral space in a rat model of IVDD and stimulating NPC regeneration. Consequently, the miR-5590-SNA@DNAgel injected in-situ gene-hydrogel microenvironment has been demonstrated to rescue the microstructure of degenerated IVDs, enhance the tissue microenvironment within the IVDs, regulate the anabolic equilibrium of the ECM in NPCs in vivo, and impede the pathogenesis of IVDD. The utilization of the gene-hydrogel microenvironment presents a promising avenue for the development of novel therapeutic interventions for IVDD and holds potential for application in other pathologies where microRNAs play a contributory role in disease pathogenesis.

## Experimental section

### Preparation of the DNA hydrogel

The DNA hydrogel was prepared as follows: Deoxyribonucleic acid salts extracted from salmon testes (salmon DNA salt) was purchased from Sigma-Aldrich. The double-distilled water was then added to dissolve the DNA powder to obtain a 100 μM DNA stock solution. The tube was placed on an incubator shaker (450 rpm, 30 °C) until no solids were visible at the bottom of the tube. Each sample was divided into 50 μL aliquots, labeled, and stored at -20 °C until further use. Hydrogels were fabricated by linking circular DNA using primers and the Rolling Circle Amplification (RCA) procedure.

### Preparation of miR-5590-loaded SNA gold nanoparticles

To prepare a 0.1 M TCEP solution, 5 mg of TCEP was combined with 174 μL of 1×TBE buffer solution. The sulfhydrylated Poly-T15 DNA was then mixed with the TCEP solution in a molar ratio of 1:200 and allowed to incubate at room temperature for 2 h. Following activation of the sulfhydryl groups on the DNA, the Poly-T15 DNA was added to a NAP-5 desalting column to remove excess TCEP from the solution. This process resulted in the purification of the sulfhydryl DNA. The purified mercapto-Poly-T15 DNA was supplemented with gold nanoparticles at a molar ratio of 1:500. Following a 2-hour incubation period, a specific volume of 5 M NaCl solution was introduced to achieve a final Na^+^ concentration of 0.01 M in the entire system. The system was subsequently agitated and allowed to rest for 30 min, after which the 5 M NaCl solution was incrementally added at 30-minute intervals. Once the Na^+^ concentration in the system reached 0.5 M, the solution was allowed to stand overnight. Subsequently, the solution was transferred to a 5000 K ultrafiltration centrifuge tube, and the surplus Poly-T15 DNA was eliminated through centrifugation with 1× TB buffer solution until the filtrate no longer exhibited an excess of Poly-T15 DNA. Finally, the resulting product was dispersed in 1 × TB buffer solution and stored in a refrigerator at 4 ℃ for future utilization. Subsequently, a 100 μM concentration solution of miR-5590 was prepared (The sequence of miR-5590 was queried using the miRNA database and was sent to Shanghai Sangon Biotechnology Co., Ltd. to synthesize miR-5590 mimics for grafting.), and SNA-AuNPs were synthesized using the Au-Poly T15:miR-5590 ratio of 1:1. The resulting mixture was subsequently purified through ultrafiltration.

### Preparation of miR-5590-SNA loaded DNA hydrogel

DNA hydrogel (100 mg) was dissolved in 500 μL of deionized water, and 4 nmol of miR-5590-SNA was added to the DNA hydrogel aqueous solution and gently blown to prepare the DNA hydrogel loaded with miR-5590-SNA. The entire procedure was conducted on ice. The prepared miR-5590-SNA@DNAgel was pale red in color.

### Evaluation of the injectability of miR-5590-SNA@DNAgel

The hydrogel was aspirated using a 1-mL syringe with a 0.5 mm diameter needle and injected into a petri dish to study the injectability of the hydrogel. Images were acquired with a digital camera collection.

### Morphology investigation by SEM

After freezing at -80 °C and freeze-drying, the prepared hydrogel was gold-sputtered before examination under SEM (FEI, USA).

### Hygroscopicity of hydrogel

To investigate the hygroscopicity of the hydrogel, 1 mL of DNA hydrogel was synthesized and separated into three parts, and the dry weight (W0) was obtained after lyophilization. The hydrogels were then dropped into 10 mL of PBS solution and incubated at 37 °C. The hydrogel was removed at 10 min, 30 min, 1 h, 2 h, 4 h, and 8 h to measure its weight (Wt). The swelling ratio was determined as follows:

1 $${\rm{Swelling}}\,{\rm{ratio}}\,{\rm{ = }}\,\left( {{\rm{Wt - W0}}} \right){\rm{/W0}}\,{\rm{ \times }}\,{\rm{100\% }}{\rm{.}}$$

The same method was used to determine the swelling ratio of the miR-5590-SNA@DNAgel.

### Biodegradability of the hydrogel

To evaluate the biodegradability of the hydrogel, 1 mL of DNA hydrogel was synthesized, separated into three parts, and immediately weighed (W0). Next, 10 mL of PBS was added to each part of the hydrogel and incubated at 37 °C with slow stirring at 100 rpm. Excess PBS was removed every two days, and the remaining hydrogel was weighed (Wt). The biodegradation level was determined using Eq. (2):

2 $${\rm{Biodegradation}}\,{\rm{level}}\,{\rm{ = }}\,\left( {{\rm{Wt/W0}}} \right)\,{\rm{ \times }}\,{\rm{100\% }}{\rm{.}}$$

The same method was used to detect the in vitro degradation of miR-5590-SNA@DNAgel.

### Mechanical property tests

To assess the mechanical properties of the hydrogels, their compression capabilities were analyzed using a universal testing machine (Instron 5567, USA). Cylindrical hydrogels measuring 10 mm in height and 5 mm in diameter were fabricated for testing. Rheological tests were performed using a rheometer (MARS 60, USA) to measure the mechanical properties of the hydrogels.

### In vitro experiments

#### Isolation and culture of NPCs

This study used NPCs obtained from Sprague-Dawley (SD) rats. Under sterile conditions, the caudal vertebrae of SD rats were separated before the NP was extracted and incubated with collagenase type II for 1 h at 37 °C. To remove tissue debris, 70-μm filters were used, and collagenase was centrifuged after incubation. NPCs were cultured in Dulbecco’s modified eagle medium (DMEM) containing 10% fetal bovine serum (Sigma) and 1% penicillin-streptomycin at 37 °C in a humidified incubator with 5% CO_2_ and 95% air. Cells from passages 2–4 were used in the experiments.

#### CCK-8 test

Hydrogels (1mL) were divided into three parts and soaked in DMEM for 24, 48 or 72 h. At the predetermined time points, the medium was replaced with CCK-8 mixture solution (10% CCK-8 solution and 90% DMEM medium), incubated for another 2 h, and then transferred to a new plate (96-well). Absorbance was measured at the wave of 450 nm by a microplate reader (BioTek microplate reader). Three replicates of each experiment were conducted.

#### Live-dead assay

A commercially available kit (LIVE/DEAD® viability/cytotoxicity kit, Invitrogen) was used to perform the live-dead assay. Briefly, NPCs (1 × 10^5^/well) were co-cultured with miR-5590-SNA and miR-5590-SNA@DNAgel for 24 h before live-dead staining. Calcein AM was used at 2 μM, and EthD-1 was used at 4 μM. A fluorescence microscope was used to verify the cell viability after 30 min of incubation at room temperature.

#### Analysis of cell apoptosis

Flow cytometry was used to assess cell apoptosis after double staining with Annexin V-FITC and PI (BD Biosciences, San Diego, CA, USA). NPCs were plated (1 × 10^5^ cells/well) and cultured for 48 h. Cells were first collected using EDTA-free trypsin, centrifuged at 300 × g for 5 min, then washed twice with PBS and blocked with 1% bovine serum albumin (BSA) for 30 min. Following this treatment, the cells were then labeled with binding buffer for 15 min according to the manufacturer’s instructions. A flow cytometer (Millipore, Burlington, MA) was used to analyze 500 μL suspensions of cells.

#### Cellular uptake of miR-5590-SNA

Cellular uptake of miR-5590-SNA was assessed using cy5.5-labeled SNA particles. Briefly, NPCs were cultured on coverslips and starved overnight with serum-free DMEM overnight. The cells were then incubated with cy5.5-labeled SNA particles for 30 min. The samples were then washed three times with PBS and fixed with 4% paraformaldehyde for 10 min, followed by DAPI staining for 10 min. The specimens were then examined using confocal fluorescence microscopy (CLSM, TCS SP8; Leica, Wetzlar, Germany).

#### In vivo bioluminescent imaging

All animal experimental procedures were reviewed and approved by the Animal Ethics Committee of Renji Hospital. SD rats (female, eight-week-old, Shanghai Jie Si Jie Laboratory Animal Co., Ltd.) were used for in vivo bioluminescent imaging. Briefly, 50 μL of miR-5590-SNA@DNAgel/NIR dye and miR-5590-SNA/NIR dye were injected into mouse tail sections 2/3 and 5/6, respectively. At several time points (0, 1, 3, 5, and 7 days), in vivo near-infrared fluorescence imaging was performed. The region of interest was circled in the mouse tail, and the fluorescence intensity was analyzed using live image J software.

#### Quantitative real-time PCR analysis

NPCs were plated at a density of 1 × 10^5^ cells/well and incubated for 2 days. TRIzol reagent (Invitrogen) was used to isolate total RNA, and reverse transcription reactions were performed using a PrimeScript RT reagent kit (Takara, Tokyo, Japan) for mRNA detection. Quantitative PCR (qPCR) was performed on an ABI7500 real-time PCR machine (Applied Biosystems, Foster City, CA, USA) using the ABI SyBr Green system, and the data were analyzed using QuantStudio 7 Flex. Gene expression was normalized to that of GAPDH, which was used as an internal control, using the 2^−ΔΔCT^ method. The primers used for qPCR are listed in Table S1.

#### Western blot

NPCs were plated at a density of 1 × 10^5^ cells/well and incubated for 2 days. Cell lysates were obtained by lysing the NPCs in the RIPA buffer (Beyotime Biotechnology, Shanghai, China). Sodium dodecyl sulfate-polyacrylamide gel electrophoresis gels were used to separate the proteins (10 mg), which were subsequently transferred to polyvinylidene difluoride membranes. After blocking with 5% non-fat dried milk, primary antibodies were incubated with the antibodies overnight at 4 ℃. The following primary antibodies were used: anti-p-mTOR (1:2000, ab263899, Abcam), anti-mTOR (1:1000, ab188570, Abcam), anti-MMP-3 (1:1000, ab36861, Abcam), anti-MMP-13 (1:2000, ab92536, Abcam), anti-ADAMTs-4 (1:2000, ab137332, Abcam), anti-ADAMTs-5 (1:5000, ab41037, Abcam), anti-collagen II (1:2000, ab51072, Abcam), anti-ACAN (1:5000, ab3778, Abcam), anti-cleaved caspase3 (1:5000, ab2302, Abcam), anti-p-ULK1 (1:5000, ab220909, Abcam), anti-ULK1 (1:5000, ab133766, Abcam), anti-Beclin-1 (1:5000, ab207612, Abcam), anti-p62 (1:5000, ab207305, Abcam), anti-LC3 (1:5000, ab192890, Abcam), and anti-GAPDH (1:5000, no.3700, Cell Signalling Technology). Tris-buffered saline supplemented with 0.05% Tween 20 was used to wash the membranes three times, followed by incubation with 1:2000 dilutions of anti-mouse (1:5000, no.7076, Cell Signalling Technology) and anti-rabbit (1:5000, no.7074, Cell Signaling Technology) secondary antibodies for 1 h. HRP was used to label the protein bands, which were detected by chemiluminescence. Using ImageJ software, we quantified the relative band density.

#### Immunofluorescence staining assay

To investigate the expressions of Collagen II, MMP3, and ACAN in NPCs, Immunofluorescence assay (IFA) was performed. Briefly, NPCs were plated at a density of 1 × 10^5^ cells/well and incubated for 2 days. Thereafter, a subsequent 4% paraformaldehyde fixation, permeabilization with Triton X-100, and blocking with 5% normal goat serum for 1 h followed. Next, cells were incubated with the primary antibodies (AF0135 for Collagen II, Affinity; ab52915 for MMP3, Abcam; NB600-504 for ACAN, NOVUS) overnight at 4 °C. The corresponding secondary antibodies were applied for 1 h. After counterstaining with DAPI for 15 min, the cells were observed and captured using CLSM.

### In vivo evaluation

#### Animal studies

The rat caudal intervertebral degeneration model was developed using 15 rats weighing 300–350 g. Briefly, the IVDs (C5-6, C6-7, C7-8, and C8-9) in the cervical spinal region were punctured using 18 G needles after anesthesia under X-ray guidance. The needles remained inserted for 30 s before being removed after puncturing the disc centers in the IVDs. Subsequently, 50 μL of the material (PBS, miR-5590-SNA, DNA hydrogel, or miR-5590-SNA@DNAgel) was injected with a 1-mL injector into the IVD. All surgical procedures were performed under aseptic conditions. All rats were housed under identical conditions and provided water and food ad libitum. Animals were housed at a temperature of 22–24 °C with a 12/12 h light/dark cycle.

#### Radiology evaluation

X-ray data were recorded at 4- and 8-weeks post-surgery. Lateral films were used to calculate the disc heights. At 8-weeks post-surgery, the experimental rats underwent MRI scans. Based on the internal gray-level of the IVD in the lumbar sagittal T2W1 MRI, we calculated the water content of the IVDs. The experimental parameters were set for the MRI according to the preliminary result: slew rate, 150 mT/m/ms; gradient field intensity, 30 mT/m. The following parameters were used for spin-echo sequence T2W1: repetition time (TR) = 3500 ms; echo time (TE) = 120 ms; scan matrix, 256 × 256; reconstruction matrix, 512 × 512; field of view (FOV) = 100.00 mm; reduced FOV (RFOV) = 100.00%; slice thickness = 3 mm; scan resolution = 0.3 mm.

#### Histological evaluation

Under sterile conditions, samples were collected 4- and 8-weeks post-surgery. After 48 h of fixation with 4% paraformaldehyde at 4 °C, the samples were decalcified for four weeks using EDTA. Decalcified samples were rinsed with tap water overnight, dehydrated with graded ethanol, vitrified with dimethylbenzene, and embedded in paraffin before freezing at -20 °C for 12 h. A Leica histo-cryotome was used to section the samples into 5 mm slices, and sections were baked for another 12 h at 60 °C. Changes in IVD structure were compared by H&E staining. Collagen remodeling and composition were observed by staining with saffron O. Quantitative analysis of Collagen II, ACAN and MMP-3 expression was determined using immunohistochemistry.

### Statistical analysis

Data are presented as the mean ± standard deviation of three or more independent replicates. Differences between groups were assessed using a one-way analysis of variance (ANOVA). P-values < 0.05 were considered statistically significant.

### Electronic supplementary material

Below is the link to the electronic supplementary material.

**Supplementary Material 1: Table S1**. Primers used in this assay

## Data Availability

All data generated or analyzed during this study are included in this article.
