# Supplementary material for: Programmable DNA hydrogel provides suitable microenvironment for enhancing autophagy-based therapies in intervertebral disc degeneration treatment
Source: J Nanobiotechnology. 2023 Sep 28;21:350. doi: 10.1186/s12951-023-02109-5 (PMC10537074; doi:10.1186/s12951-023-02109-5)
Supplement: Supplementary file 1 — Supplementary Material 1: Table S1. Primers used in this assay [file 12951_2023_2109_MOESM1_ESM.docx]

**Programmable DNA hydrogel provides suitable microenvironment for enhancing autophagy-based therapies in intervertebral disc degeneration treatment**

*Song Qingxin,^a^ Jiang Kai,^b^ Zheng Dandan,^a^ Jin Linyu,^c^ Chen Xiuyuan,^a^ Feng Yubo,^a^ Wang Kun,^a^ Han Yingchao,^a^ Chen Hao,^a^ Song Jie,^d,e,^** *Chen Zhi,^a,^** *Shen Hongxing ^a,^**

^a^ Department of Spine Surgery Renji Hospital Shanghai JiaoTong University School of Medicine 160 Pujian Road, Shanghai 200127, P. R. China

^b^ Department of Ophthalmology and Vision Science, Shanghai Eye, Ear, Nose and Throat Hospital, Fudan University, Shanghai, China

^c^ Department of Orthopedics, Shanghai Key Laboratory for Prevention and Treatment of Bone and Joint Diseases, Shanghai Institute of Traumatology and Orthopedics, Ruijin Hospital, Shanghai Jiaotong University School of Medicine, Shanghai, P. R. China

^d^ Department of Instrument Science and Engineering, School of Electronic Information and Electrical Engineering, Shanghai Jiao Tong University, Shanghai 200240, China

^e^ The Cancer Hospital of the University of Chinese Academy of Sciences, Hangzhou, Zhejiang, 310022, China.

*Corresponding authors:

E-mail: shenhxgk@126.com (Shen Hongxing), mcgrady923@126.com (Chen Zhi), sjie@sjtu.edu.cn (Song Jie)

Song Qingxin, Jiang Kai, and Zheng Dandan contributed equally to this work.

**Table S1** Primers used in this assay

| Gene and primer direction | Primer sequence (5′to 3′) |
| --- | --- |
| R-U6 |  |
| Forward | CGCTTCGGCAGCACATATACT |
| Reverse | AACGCTTCACGAATTTGCGT |
| miR-5590-3p |  |
| Forward | GGGCCCAATAAAGTTCATGT |
| Reverse | CTCAACTGGTGTCGTGGAGTC |
| R-GAPDH |  |
| Forward | AACAGCAACTCCCATTCTTCC |
| Reverse | TGGTCCAGGGTTTCTTACTCC |
| R-DDX5 |  |
| Forward | TTATTCGTCTGATGGAAGAGATC |
| Reverse | TCGGTAGCAATCAGAATAGGAG |
| R-Bax |  |
| Forward | TGAACTGGACAACAACATGGAG |
| Reverse | AGCAAAGTAGAAAAGGGCAACC |
| R-Bcl-2 |  |
| Forward | TTGTGGCCTTCTTTGAGTTCG |
| Reverse | TTCAGAGACAGCCAGGAGAAATC |
| R-MTOR |  |
| Forward | GATGAGTCAGGAGGAGTCTACTCG |
| Reverse | GGATCACTTGAGGGGAGGAG |
| R-MMP3 |  |
| Forward | GGTGGCTCTCCTTGTCATTTTC |
| Reverse | AGGTTGAGCAGGTAGGTATCCG |
| R-MMP13 |  |
| Forward | GCAGCTCCAAAGGCTACAACTT |
| Reverse | GTAATGGCATCAAGGGATAGGG |
| Collagen II |  |
| Forward | CAGAGTGGAAGAGCGGAGACTAC |
| Reverse | GCCCTCAGTGGACAGTAGACG |
| R-ACAN |  |
| Forward | CAAACAGCAGAAACAGCCAAGT |
| Reverse | GAAGGCATAAGCATGTGAAAGTG |
| R-ADAMTS4 |  |
| Forward | GTGGTGACTCGTCTAGTAATCCT |
| Reverse | GTGGTCAGGATCTGAGTCGTTC |
| R-ADAMTS5 |  |
| Forward | GATCACAGAATTTCTGGATGACG |
| Reverse | TCTTGGTCAGACACACCATTTG |
